# Did Michelangelo paint a young adult woman with breast cancer in “The Flood” (Sistine Chapel, Rome)?

**DOI:** 10.1016/j.breast.2024.103823

**Published:** 2024-10-19

**Authors:** Andreas G. Nerlich, Johann C. Dewaal, Antonio Perciaccante, Laura Cortesi, Serena Di Cosimo, Judith Wimmer, Simon T. Donell, Raffaella Bianucci

**Affiliations:** a Institute of Legal Medicine, Department of Forensic Histology, Paleopathology and Mummy Research, Ludwig-Maximilians-University, Munich, Germany; b Breast Center, Dachau, Germany; c Azienda Sanitaria Universitaria Giuliano Isontina, Department of Medicine, “San Giovanni di Dio” Hospital, Gorizia, Italy; d Laboratory Anthropology, Archaeology, Biology (LAAB), Paris-Saclay University, Montigny-Le-Bretonneux, France; e Fondazione IRCCS Istituto Nazionale Dei Tumori, Milano, Italy; f Modena Hospital University, Modena, Italy; g Department of Art and Heritage Conservation, Diocese Linz, Linz, Austria; h Norwich Medical School, University of East Anglia, Norwich, UK

**Keywords:** Iconodiagnosis, Breast disease, Michelangelo, Sistine Chapel, Bible, Symbolism

## Abstract

*The Flood* is the first pictorial scene that Michelangelo Buonarroti painted on the ceiling of the Sistine Chapel in the Vatican. On the right side of the fresco a woman with abnormal breast morphology is presented and the nature of her disease is considered using the Guidelines for Iconodiagnosis. A team of experts covering art history, art expertise, medicine, genetics, and pathology undertook the process and concluded that the pathology shown is probably breast cancer, most likely linked to the symbolic significance of an inevitable death as expressed in the Book of Genesis.

## Highlights

• Guidelines for Iconodiagnosis are used to diagnose breast cancer in a fresco (*The Flood)* by Michelangelo.
• Pathological features, remindful of modern-day breast carcinoma, are present.
• Breast cancer is linked to the impermanence of life and punishment.

## 1 Introduction

The breast, as a symbol of motherhood and femininity, has consistently held a significant role in artistic representations. Occasionally breast pathology can be identified in sculptures and paintings [[1], [2], [3], [4], [5]]. This has sparked debate among scholars about whether these abnormalities were intentionally depicted by the artists and, if so, what message they intended to convey to their patrons and the public [[6], [7], [8], [9]]. The process of analyzing and interpreting these potential medical conditions in art is known as iconodiagnosis which requires collaboration among experts in biomedicine, medical history, and art history [10]. Here we report the case of a breast anomaly, painted by Michelangelo and include a state-of-the-art discussion on iconodiagnosis of this fresco to investigate whether this represents breast cancer (BC).

## 2 Material and methods

**Fig. 1:**
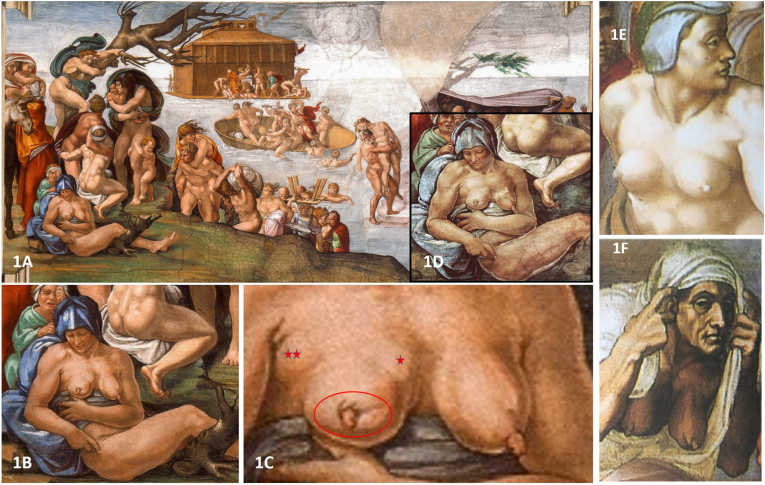
In the second span of the vault of the Sistine Chapel, Michelangelo and his collaborators represented *The Flood*. ***1A:*** The biblical event was depicted by Michelangelo between July and August 1508 [11]; ***1B:*** Detail of the vault scene showing an almost naked woman. Her bare breasts are presented; ***1C:*** Close up of the right breast shows nipple retraction, fissural defects especially of the upper and medial areola (circle). At the upper medial quadrant (one star) and towards the axilla (two stars) small bulging lumps are seen (Public domain available at: https://www.wga.hu/html_m/m/michelan/3sistina/1genesis/2flood/02_3ce2.html; ***1D:*** Pre-restoration image showing very similar features as in 1B (Reproduced with permission of Scala Archives, Firenze, Italy); ***1E:*** Young woman with healthy breasts depicted on the right side of the Last Judgement in the Sistine Chapel. The breasts are firm and symmetrically positioned, with a rounded and youthful appearance, seeming almost sculpted against the chest. The nipples are normal in size and shape, though one subtly points to the right and the other to the left, a condition known as "lateralization" or "nipple asymmetry." Despite this small variation, they appear balanced and harmonious. The way they sit on the chest suggests the artist's distinctive habit of depicting female breasts on a male torso, emphasizing the anatomical contrast and a certain artistic exaggeration, as if resting more on the chest wall than projecting forward [Public domain available at: https://en.wikipedia.org/wiki/File:Last_Judgement_(Michelangelo).jpg]; ***1 F:*** Old woman with healthy breasts depicted in the upper right side of the Last Judgement (Sistine Chapel) The breasts show the natural effects of aging, sagging symmetrically under the pull of gravity. The skin is looser, and the breasts droop toward the lower chest. The nipples are ptotic (a condition where the nipples point downward due to breast sagging) and reflect the advanced age of the person. Despite this, the breasts retain a natural appearance, still within a range of normal aging without pathological signs. The overall impression matches the aged features of the person's face, creating a consistent portrayal of time's effects on the body [Public domain available at: https://en.wikipedia.org/wiki/File:Last_Judgement_(Michelangelo).jpg].

In the year 1508, by the order of Pope Julius II (1443–1513), Michelangelo Buonarroti started to paint the vault of the Sistine Chapel [11] with approximately 300 figures. The principal theme is the story of the Genesis in the Old Testament, presented in a unique flowing composition. In the second span of the vault, the first pictorial scene was “*The Flood”* (https://www.vatican.va/archive/bible/genesis/documents/bible_genesis_en.html) where the artist painted a group of individuals that are fleeing from the rising water. On the left side of the fresco, an almost naked young adult woman is depicted. She wears only a blue headscarf, indicating her married status, and a blue cloak. The fresco was analyzed following the recently established guidelines for a correct approach to the diagnosis of medical conditions in works of art. Diagnosis of exclusion was performed and an estimation of the “Level of Evidence” for the diagnosis was proposed and agreed. The presence or absence of any kind of artifacts pre- (Fig. 1D) and post-restoration (Fig. 1B), was evaluated [10]. Last, but not least, the theological meaning of this particular representation was also considered [10].

## 3 Results

Careful observation indicated that her left breast shows age or breastfeeding-associated ptosis with a prominent nipple and smooth contours (Figs.1 B/1C). The contrast with the right breast is evident. Although slightly elevated by her right arm, there is a significantly retracted and deformed nipple. The areolar/periareolar skin is retracted, the medial part of the areola seems eroded, the skin cranial to the nipple is deeply indented and scar-like retracted. No overt ulcer is depicted. The upper medial quadrant shows a slight bulge consistent with a lump. Similarly, towards the left axilla, another slight bulge is seen, which could represent enlarged nodes. The medial side of the breast appears slightly discolored representing an artistic effect rather than a typical *peau d′orange*.

Comparison with a pre-restoration photograph (Fig. 1D) showed that the shape of the original breasts did not result in substantial changes [11]. Some might argue that the woman depicted is quite young for a diagnosis of BC, given that today 85 % of patients with this disease are over 50. However, applying modern data to the Renaissance period is not entirely accurate, as the average life expectancy then was around 35 years, which could have influenced the presentation and characteristics of cancer at that time. During the Renaissance, epidemics were common, including tuberculosis, which is known to spread to the breast via lymphatic or blood routes. However, the nodular form of tuberculous mastitis, which aligns with the clinical presentation here, is uncommon even in regions where tuberculosis is endemic. It seems less plausible to consider puerperal mastitis, given the partial involvement of the breast and the lack of signs of breastfeeding. Plasma cell mastitis, a chronic condition typically seen in older women, is even less likely due to the apparent young age of the subject [[12], [13], [14]]. Also trauma, chronic inflammatory diseases like lupus mastitis and sarcoidosis, as well as endocrinopathies such as diabetes, can present with breast abnormalities, although less commonly. Some of these conditions may exhibit distinctive pathognomonic signs that facilitate diagnosis, while others closely mimic primary breast cancer, making the diagnosis more challenging and requiring a complete clinical history and relevant exams for accurate assessment [15].

## 4 Discussion

Here we report for the first time that the right breast in Michelangelo's *“The Flood”* shows features consistent with breast carcinoma. The retrospective diagnosis of suspected breast cancer in the woman depicted in *“The Flood”* was performed after having carefully observed several naked breasts depicted and sculpted by Michelangelo including naked breasts depicted in the Sistine Chapel *(“The Last Judgement”*) and the statues of the”Dawn” and the “Night” (Cappelle Medicee, Florence).

The result of our observation indicates that Michelangelo had knowledge of healthy breasts of different sizes and morphologies and adapted them to the biblical feminine personages (Fig. 1E/1F). Reproduction of pathologic breast conditions with a specific symbolism or theological meaning was purposedly represented by the artist.

In the present case, both the identity and history of the woman with a pathological breast is are irrelevant. Michelangelo did not resort to a living model (or living models either male or female) when depicting the story of the Genesis. No realistic portraits are presented in the scene. It is rather the composition of an idea based upon the story of the Bible with idealised faces and bodies.

Saunders [16] suggested that, during the Renaissance, feminine figures in art were often modeled after males, as respectable women would not pose naked. Thus, Michelangelo's women might be “men with breasts,” with heads and breasts simply placed on male figures. Although male models were commonly used, many artists from 1500 CE onwards did employ female models. Additionally, the Renaissance celebrated aesthetic androgyny, a gender ambiguity seen in allegorical figures by Michelangelo, Titian, and Vasari [17].

Michelangelo was a sculptor and the Sistine project was his first monumental painting (and the first in fresco technique). This tenor of his work is the reason why his artistic style is highly three-dimensional and rich in contrast. As a result, his human beings, women included, are drawn as very muscular individuals following the canon of aesthetic androgyny. Ordinary people did not look this way. To intensify the contrast to this ideal, the bad could be marked by an anomaly/pathology (deformities, illness, distorted facial expression) so that the viewer could “read” the difference between the righteous and the evil.

Considering these stylistic factors, the comparison of the right and left breast may suggest a pathological condition. Michelangelo, who began assisting to autopsies when 17 years old [18], would have observed pathology potentially including BC. BC onset at a very young age could be associated with an inheritance of germline pathogenic variants (gPV) in autosomal penetrance genes. Of interest, the *BRCA1*c.4096+1G > A and the *BRCA1* 1499insA gPV have been identified as founder mutations from 1800 years ago in the Tuscany region and subsequently disseminated [19,20]. Furthermore, *BRCA1* gPV are associated to rapidly increasing BC at a very young age, accounting for about 5–10 % of all BC and 13 % of triple negative BC, with a 42 years median age at onset compared to 56 years in the general population Another interpretation could be the presence of a “*de novo*” gPV, mostly related to young age of BC onset [21]. The theological meaning is important too, since the Ashkenazi Jew population harbors three different founder mutations in 1 % of people, reinforcing the concept of the Jewish punishment in the Book of Genesis. However, due to the reduction in life expectancy of the Renaissance period, these inherited cancers could be less expressed than found currently.

Despite Gelinsky's challenge [9], previous publications suggest that Michelangelo's sculpture *“The Night”* (Cappelle Medicee, Florence) might represent breast cancer, a theme later explored by Michele di Ridolfo del Ghirlandaio [2].

Given that 14 years separated Michelangelo's fresco in the Sistine Chapel from his later sculptural work *(“The Night”*), it is plausible that the artist was influenced by the features of unhealthy breasts. Also *“The Night”* was not sculpted resorting to a living model. She is the idealisation of an adult mature woman who becomes the symbol for (eternal) sleep, death, darkness, memory loss. In her case, the representation of a possible breast cancer is linked to fate, not to punishment.

The theological motivation for the Flood is obvious: “And GOD saw that the wickedness of man was great in the earth, and that every imagination of the thoughts of his heart was only evil continually.” (Gen. 6,5)(https://www.biblegateway.com/passage/?search=Genesis%206&version=NKJV). The righteous found a safe refuge inside the Ark (on the backfront of the fresco). The remaining individuals are punished and will die. (https://www.biblegateway.com/passage/?search=Genesis%207&version=NKJV). More specifically, within the group of persons fleeing the Flood, there are a few types of individuals who “represent” the seven deadly sins: gluttony and sloth (the man with the barrel), anger (the people fighting in the boat), covetousness (the woman carrying her household assets). All these details indicate the reasons for their punishment. Perhaps BC may represent a personal punishment for lust (?).

The representation of a probable breast cancer is linked to the concept of the impermanence of life and has the significance of punishment. It shall also be noted that the woman is pointing to the ground with the forefinger of her right hand (the side where she lifts and exhibits her breast). This gesture may refer to Genesis 3,19: " … for dust you are and to dust you will return." Both her incurable illness and the gesture are inseparable and illustrate mortality due to punishment. Furthermore, there is a theological parallel since Gen 3,19 yet reveals God's punishment for sinning. Death (and being aware of the end) is the final consequence of Adam's sin. In accordance with this verse from the Bible, the cropped tree under the legs could remind us of the tree of knowledge.

As an expression of Neoplatonism, by which Michelangelo was influenced, the pursuit of beauty and harmony could lead to immortality, whereas physical disfiguration or illness was an expression of spiritual abyss. This metaphor shall be taken into consideration when the woman lifts up her breast so that it can be clearly observed by the viewer. Michelangelo's individuals are painfully aware of their destiny rather than being petrified by the danger that hovers over them [11].

In conclusion, Michelangelo's depiction in *“The Flood”* suggests characteristics of breast cancer (Level of Evidence I) [10]. The evidence of the pathology is fully corroborated by the symbolism and the theological meaning underlying this representation of life and death.

## CRediT authorship contribution statement

**Andreas G. Nerlich:** Writing – review & editing, Writing – original draft, Validation, Supervision, Methodology, Investigation, Data curation, Conceptualization. **Johann C. Dewaal:** Writing – review & editing, Writing – original draft, Investigation, Data curation. **Antonio Perciaccante:** Writing – review & editing, Writing – original draft, Methodology, Investigation. **Laura Cortesi:** Writing – review & editing. **Serena Di Cosimo:** Writing – review & editing, Methodology, Investigation. **Judith Wimmer:** Writing – review & editing, Methodology, Data curation. **Simon T. Donell:** Writing – review & editing, Writing – original draft, Methodology, Investigation, Formal analysis, Data curation. **Raffaella Bianucci:** Writing – review & editing, Writing – original draft, Supervision, Methodology, Investigation, Formal analysis, Data curation, Conceptualization.
